# Clinical and tomographical evaluation of periodontal phenotype among undergraduate dental students in India

**DOI:** 10.6026/973206300213536

**Published:** 2025-10-31

**Authors:** Kavita Lohiya, Hiroj Bagde, Meenakshi Boddun, Aishwarya Duble, Neha Sahu, Sonal Kumbare

**Affiliations:** 1 Department of Periodontology and Implantology, Chhattisgarh Dental College and Research Institute, Sundra Rajnandgaon, Chhattisgarh, India; 2 Department of Periodontics, Government Dental College, Raipur, Chhattisgarh, India

**Keywords:** Periodontal phenotype, CBCT, gingival thickness, bone thickness, keratinized tissue

## Abstract

Periodontal biotypes in 53 healthy dental students were evaluated using cone-beam computed tomography (CBCT). Gingival thickness (GT), bone thickness (BT) and width of keratinized tissue (WKT) were measured in the upper and lower anterior teeth. The thick scalloped biotype was most prevalent (60%), followed by the thin scalloped (30%) and thick flat scalloped (10%) types. Bone thickness was consistently greater than gingival thickness, with the thick-flat biotype showing the highest WKT. Thus, the mean biological width was 2.02 mm, indicating predominantly thick biotypes with thinner overlying gingiva.

## Background:

Patients with thin gingival thickness often require additional surgical procedures, while those with thicker tissues can usually be treated with simpler techniques; therefore, determining the tissue biotype before restorative treatment is essential, as the thickness of gingival and bone tissues affects outcomes, likely due to variations in blood supply to the underlying bone and susceptibility to resorption [1]. Gingival thickness, keratinized tissue width and bone morphotype are key parameters for classifying biotypes and play a significant role in the development or progression of mucogingival defects, with the assessment of surrounding soft and hard tissues being the most critical factor for the success of periodontal and restorative treatments [2]. The term "gingival biotype" is often used to describe the thickness of the gingiva in the buccolingual direction. However, other terms like "periodontal biotype," "periodontal morphotype", "gingival morphotype," and "gingival phenotype" are also used. These terms encompass not only differences in gingival thickness (GT) and width of keratinized tissue (KTW), but also include features such as bone structure, tooth shape and the overall form of the gingiva and surrounding periodontium [3]. A thin periodontal phenotype is usually linked to poor healing after surgery and often requires extra procedures, while thicker tissues allow for simpler treatment [4]. Various invasive and noninvasive methods are used to assess periodontal phenotype (PP), including the direct method, periodontal probe transparency (TRAN), ultrasound devices and CBCT imaging [5]. Measuring facial soft tissue thickness is crucial for accurate periodontal treatment planning and predicting procedural success, but traditional intraoral X rays, despite being commonly used, lack three-dimensional visualization, which limits the detection of subtle defects, underestimates bone loss and often misses critical anatomical details necessary for comprehensive diagnosis [6, 7 and 8]. Cone-beam computed tomography (CBCT) is a recent advancement in dental and maxillofacial diagnostic imaging that provides cost-effective, lower-radiation, three-dimensional visualization of craniofacial structures by using a cone-shaped X-ray beam instead of the fan-shaped beam used in conventional computed tomography (CT) [9, 10]. In a 2008 study by Alessandro *et al.*, a new method called soft tissue CBCT (ST CBCT) was introduced, based on the CBCT technique which allows clear viewing and measurement of both hard and soft tissue distances making it simple and noninvasive method, for dentists to understand the relationship between different parts of the periodontium [11]. Thin periodontal phenotype (PP) is often linked to bone problems and gum recession, especially after surgery or orthodontic treatment. The shape and thickness of the gums in the upper front teeth area are important for achieving good aesthetic results. Understanding the periodontal biotype is essential because features like gum thickness, gum width and bone shape affect how the tissues respond to injury, infections, or dental treatments like implants or braces. Therefore, it is of interest to assess the periodontal phenotype of undergraduate dental students using both clinical exams and CBCT scans among Indians.

## Methodology:

A proposed study was conducted on 53 undergraduate dental students from the Department of Periodontology and Implantology, Chhattisgarh Dental College & Research Institute, Rajnandgaon, to evaluate periodontal phenotype using both clinical and radiographic methods. Out of the total participants, a convenience sample of 20 students was selected for detailed CBCT evaluation of gingival and bone thickness. The study population was divided into two groups based on the teeth evaluated: Group 1 consisted of the upper anterior segment (central incisors, lateral incisors and canines on both sides), while Group 2 included the lower anterior segment (central incisors, lateral incisors and canines on both sides). Clinical evaluation was done using two classifications first one as thin scalloped phenotype, Thick flat phenotype, Thick scalloped phenotype given by Zweers, (2014) ans second one involved measuring gingival thickness (GT) using a University of North Carolina 15 Hu Friedy periodontal probe by checking probe visibility through the free buccal gingiva. Based on this transparency method, gingiva was classified as thin (≤ 1.0 mm, probe visible) or thick (≥ 1.0 mm, probe not visible) categorized according to De Rouck *et al.*,(2009). Radiographic evaluation of buccal bone thickness was performed using Cone Beam Computed Tomography (NEWTOM CBCT, Italy) with NNT Viewer in house software. Standardization during scans was ensured using an acrylic lip retractor to maintain proper exposure of the gingiva and teeth. Measurements of bone and gingival thickness were taken at predefined levels along the anterior teeth. Ethical clearance was obtained from the Institutional Ethical Committee and participants were informed about the study in their regional language, with written informed consent collected prior to the procedure. The inclusion criteria comprised healthy undergraduate students aged ≥18 years with a clinically healthy periodontium. The exclusion criteria included a history of antibiotic therapy in the last six months, systemic conditions like diabetes or hypertension, endo perio lesions, prior orthodontic or periodontal treatment in the anterior region, presence of prostheses, smoking, alcohol consumption, or any medical compromise. This methodology ensured a comprehensive assessment of periodontal phenotype by combining clinical examination and CBCT imaging, allowing for accurate evaluation of gingival and bone thickness across different anterior teeth.

## Statistical analysis:

The collected data were entered into a Microsoft Excel spreadsheet and subsequently analyzed using the Statistical Package for the Social Sciences (SPSS) software, version 20.0 for Windows (IBM Corp., Armonk, NY, USA). Descriptive statistics were used to summarize the data. Continuous variables were expressed as mean ± standard deviation (SD), while categorical variables were presented as frequencies and percentages. Prior to analysis, the data were assessed for normality using appropriate statistical tests. For continuous variables that were normally distributed, comparisons between groups were performed using the independent samples t-test. For non-normally distributed continuous variables, the Mann-Whitney U test was used. Categorical variables were analyzed using the Chi-square test or Fisher's exact test, based on the distribution of expected frequencies. A p-value of less than 0.05 was considered statistically significant. The alpha error was set at 5% with a 95% confidence interval and the power of the study was maintained at 80%, corresponding to a beta error of 20%.

## Results:

The analysis of periodontal phenotype was conducted separately for the upper and lower anterior segments using two classification systems. According to the first classification, in the upper anterior segment, 3 subjects (30%) exhibited a thin, scalloped gingival phenotype. The majority of participants, 6 subjects (60%), demonstrated a thick scalloped phenotype, while only 1 subject (10%) was found to have a thick flat gingival phenotype as described in Figure 1a (see PDF). A similar distribution was observed in the lower anterior segment, where 3 subjects (30%) had a thin scalloped phenotype, 6 subjects (60%) presented with a thick scalloped phenotype and 1 subject (10%) showed a thick flat phenotype illustrated in Figure 1b (see PDF). This consistency across both arches indicates that the thick scalloped phenotype was the most prevalent among the study population in both upper and lower anterior segments as shown in Figure 1c (see PDF). In the second classification of periodontal phenotype, slight variations were noted. In the upper anterior segment, 3 subjects (30%) still exhibited the thin scalloped phenotype, whereas the majority, 7 subjects (70%), demonstrated a thick scalloped phenotype. In the lower anterior segment, the distribution remained similar to the first classification, with 3 subjects (30%) having a thin scalloped phenotype and 6 subjects (60%) were exhibiting a thick scalloped phenotype. These findings reaffirm the dominance of the thick scalloped phenotype in the study population, particularly in the anterior segments of both the upper and lower arches as summarised in Figure 2 (see PDF). Figure 3 (see PDF) describes the width of keratinised tissue (KTT) and showed a consistent trend across all tooth positions. The upper arch demonstrated a greater width of keratinised tissue compared to the lower arch. The average width in the upper arch was 5.43 mm, which was slightly higher than the average width in the lower arch, recorded at 5.21 mm. This suggests a generally more robust band of keratinised gingiva in the upper anterior region. Bone thickness measurements revealed that the highest thickness was consistently observed at the region extending from the cemento-enamel junction to the bone crest (CEJ-BC) in both the upper and lower arches. This was followed in descending order by thickness at BT1, BT2, GT1 and GT3, respectively. This pattern remained consistent across both arches, indicating that the greatest bone density is found near the crest, with gradual thinning toward the apical portions of the gingival tissue and bone elaborated as in Figure 4 (see PDF). Overall, the study highlighted the predominance of the thick scalloped periodontal phenotype among undergraduate dental students and emphasized anatomical differences in soft and hard tissue characteristics between the upper and lower anterior segments. These findings could have implications for clinical decision-making in periodontal and restorative procedures.

## Discussion:

Different periodontal biotypes influence the clinical results of therapeutic procedures in various ways. Assessing these biotypes aids in predicting treatment outcomes, as the stability of the alveolar crest and the location of the free gingival margin are closely related to the thickness of both the bone and gingival tissues [12]. According to the both the classification [13, 14], the thick scalloped phenotype was the most prevalent among the study population in both upper and lower anterior segments. Thick-scalloped PPs were the most common (60%), followed by thin scalloped (30%) and thick-flat scalloped (10%). Diverseness exist in literature regarding the distribution of gingival biotype in maxilla and mandible, with Cuny-Houchmand *et al.* [15] report of thick gingival biotype in maxilla and Pascual *et al.* [16] conclusion that of soft and hard tissue dimensions of anterior teeth in both the arches are commensurable. Other studies, including those by Shah *et al.* found no significant association between sex and gingival thickness in the maxillary anterior region [17]. Lang and Loe claimed that 2 mm of keratinized gingiva and 1 mm of attached gingiva are required for gingival health which was in similar comparison with the result of present study i.e., Width of keratinized tissue for upper arch was more as compared to lower arch at each teeth position [18]. Bouri *et al.* [19] evaluated the relationship between the width of keratinized mucosa and the peri-implant soft tissue health, finding that a greater width of keratinized gingiva around dental implants correlates with reduced mean bone resorption and better soft tissue parameters in the following study [20]. CBCT showed high accuracy in measuring gingival thickness (GT), with only minor variations compared to the transgingival technique. Thin gingival phenotypes were found to be positively associated with the occurrence of dehiscence and fenestration defects, this trend was consistent in both arches, showing that bone density is highest near the crest and gradually decreases toward the apical regions of the gingival tissue and bone.

## Source of Support and Funding:

Self-Funded

## Conclusion:

CBCT imaging demonstrates that maxillary anterior teeth commonly exhibit thin labial bone, with canines being most vulnerable to recession risk. Combining CBCT with clinical evaluation provides a more accurate assessment of periodontal phenotype for precise treatment planning. Future multicenter longitudinal studies are warranted to validate these findings and explore racial and ethnic variations in gingival thickness.
